# SARS-CoV-2 Omicron Variant in Croatia—Rapid Detection of the First Case and Cross-Border Spread

**DOI:** 10.3390/pathogens11050511

**Published:** 2022-04-26

**Authors:** Ivana Ferenčak, Mihaela Obrovac, Ljiljana Žmak, Josipa Kuzle, Goranka Petrović, Tatjana Vilibić-Čavlek, Dragan Jurić, Anita Jurić, Željka Hruškar, Krunoslav Capak, Vladimir Stevanović, Maja Milanović, Marija Govedarica, Danijela Vujošević, Irena Tabain

**Affiliations:** 1 Department of Microbiology, Croatian Institute of Public Health, 10000 Zagreb, Croatia; ivana.ferencak@hzjz.hr (I.F.); mihaela.obrovac@hzjz.hr (M.O.); ljiljana.zmak@hzjz.hr (L.Ž.); josipa.kuzle@hzjz.hr (J.K.); dragan.juric@hzjz.hr (D.J.); misicanita1@gmail.com (A.J.); zeljka.hruskar@hzjz.hr (Ž.H.); irena.tabain@hzjz.hr (I.T.); 2 Department of Microbiology, School of Medicine, University of Zagreb, 10000 Zagreb, Croatia; 3 Department of Epidemiology, Croatian Institute of Public Health, 10000 Zagreb, Croatia; goranka.petrovic@hzjz.hr; 4 Environmental Health Department, Croatian Institute of Public Health, 10000 Zagreb, Croatia; kcapak@hzjz.hr; 5 Department of Microbiology and Infectious Diseases with Clinic, Faculty of Veterinary Medicine, University of Zagreb, 10000 Zagreb, Croatia; vladostevanovic@gmail.com; 6 Department of Epidemiology, Institute of Public Health, 81110 Podgorica, Montenegro; maja.milanovic@ijzcg.me (M.M.); marija.govedarica@ijzcg.me (M.G.); danijela.vujosevic@ijzcg.me (D.V.)

**Keywords:** SARS-CoV-2, Omicron variant, whole-genome sequencing, surveillance, Croatia

## Abstract

Background: Due to rapid spread, the Omicron variant has become the dominant SARS-CoV-2 variant responsible for infections worldwide. We present the first detection of the Omicron variant in Croatia which resulted in rapid cross-border spreading. Methods: Whole-genome sequencing was performed using the Illumina MiniSeq sequencing system. SARS-CoV-2 lineages were identified using the PANGOLIN and GISAID databases. Results: The first case of the Omicron variant (BA.1.17) emerged in Croatia after a workshop held in Zagreb in November 2021. The patient reported a history of previous COVID-19 and received two doses of an mRNA vaccine. Three additional cases were detected among Croatian participants of the workshop. At the beginning of December, SARS-CoV-2 infection was confirmed in one participant from Montenegro and her husband. Phylogenetic analysis showed that the detected Omicron variants were closely related to the first Croatian case, confirming the connection with the workshop outbreak and rapid cross-border spreading. Subsequent analyses of SARS-CoV-2 positive samples in Croatia showed the rapid introduction of the Omicron variant and depletion of the Delta variant resulting in the fifth pandemic wave. Conclusions: Genomic monitoring and early detection of novel SARS-CoV-2 variants are essential to implement timely epidemiological interventions and reduce further transmission in the population.

## 1. Introduction

During the past two years, from the beginning of the coronavirus disease (COVID-19) pandemic, the World Health Organization (WHO) categorised five severe acute respiratory syndrome coronavirus 2 (SARS-CoV-2) variants to be variants of concern (VOC) [1]. These variants possess concerning characteristics, including increased transmissibility or/and disease severity, reduction in neutralisation by antibodies produced during previous infection or vaccination, as well as reduced effectiveness of treatments or diagnostic testing [1]. However, no variant has caused as much concern as the last variant identified on 24 November 2021, in South Africa, named Omicron, after the 15th letter in the Greek alphabet [2]. Only two days after the identification of this new variant, the WHO designated it as a VOC [1] due to a high number of mutations in the spike protein [3]. Omicron harbours around 30 mutations in the S gene, thus having 15 more alterations in the same region compared to other VOCs identified so far [4]. There has been a steep increase in COVID-19 cases in almost all provinces in South Africa [2], coinciding with the detection of this variant, forcing most countries to adopt more rigorous epidemiological measures to contain the further spread of the novel VOC [5]. The Omicron variant has spread rapidly over the globe, and, by the beginning of January 2022, researchers detected it in 149 countries, including Croatia, even in countries with a high vaccination coverage [6]. Omicron rapidly replaced the Delta variant as the dominant variant, and by week 02 of 2022, Delta constituted less than 15% of all Croatian positive samples [7]. The number of Omicron cases increased sharply because the variant evades the existing immunity [8,9] and is inherently more transmissible than previous variants [10]. Fast genomic characterisation is essential to prevent the cross-border spread of novel SARS-CoV-2 variants and implement immediate and effective epidemiologic measures [11].

We present the first case of the Omicron variant in Croatia resulting in rapid cross-border spreading. 

## 2. Results

The first suspected cases of the Omicron variant in Croatia emerged after a workshop held in Zagreb, on 25–26 November 2021, with approximately 20 participants from Croatia, Bulgaria, Albania, Montenegro, and Cyprus.

The patient suspected of being the index developed flu-like symptoms two days after the workshop and was tested in the National Reference Laboratory for Respiratory Viruses at the Croatian Institute of Public Health on 4 December 2021. The patient reported a history of previous COVID-19 and vaccination with two doses of the mRNA vaccine. 

The sample tested positive for SARS-CoV-2; thus, single nucleotide polymorphism (SNP) screening assays [12,13] were performed using commercial qPCR kits (Allplex™ SARS-CoV-2 Variants I Assay; Allplex™ SARS-CoV-2 Variants II Assay, Seegene, Seoul, Korea) [14,15]; the results obtained are shown in Table 1.

Since the SNP profile in the sample was highly indicative of the Omicron variant [16] and excluded the Delta variant [17], whole-genome sequencing (WGS) was conducted. On 6 December 2021, the isolate was confirmed to be Omicron variant (BA.1) [18,19,20], the first confirmed case in Croatia (Global Initiative on Sharing All Influenza Data database (GISAID) accession ID: EPI_ISL_7210427) [21,22,23,24]. Immediate epidemiological investigation and contact tracing were carried out, during which epidemiologists identified an additional three possible cases of the Omicron variant among Croatian participants of the workshop. All the case participants were fully vaccinated against COVID-19. All three patients tested positive by both RT-PCR and SNP analysis. WGS was performed, also confirming the Omicron variant (GISAID accession IDs: EPI_ISL_7635857, EPI_ISL_7892496 and EPI_ISL_7897799) [21,22,23,24].

On 6 December 2021, in Montenegro, one participant of the workshop and her husband were strongly suspected of having the Omicron variant by screening RT-PCR test [25], exhibiting S gene target failure due to the presence of deletion HV69/70 [26], which was confirmed by WGS performed in a commercial laboratory (GISAID accession IDs: EPI_ISL_8366166, EPI_ISL_8365856) [21,22,23,24].

**Figure 1:**
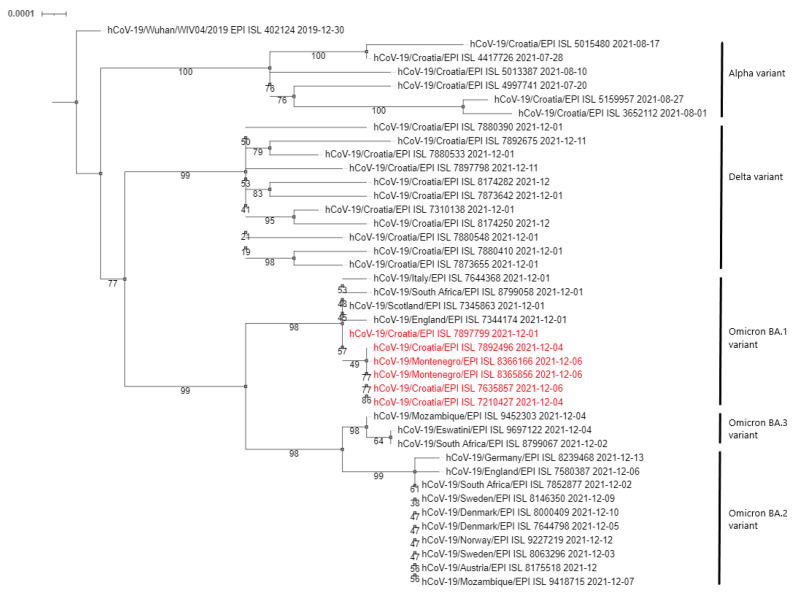
Phylogenetic neighbour-joining analysis of the whole genome sequenced SARS-CoV-2 viruses. Strains detected in this outbreak are marked red. Country of origin, GISAID Accession IDs and date of collecting samples are specified. Supporting (50%) bootstrap values of 1000 replicates are displayed at the nodes. Horizontal distances are proportional to genetic distance. The scale bar indicates nucleotide substitutions per site.

Phylogenetic analysis of the sequenced samples, presented in the form of a cladogram in Figure 1, showed that the Omicron variants detected in the Montenegro patients were closely related to the first Croatian case, confirming the connection with the Zagreb workshop outbreak and rapid cross-border infection.

The available demographic characteristics of patients collected through epidemiologic investigations, accession IDs of their sequenced samples [21,22,23,24] and assigned lineages [18,19,20,27] are presented in Table 2.

**Figure 2:**
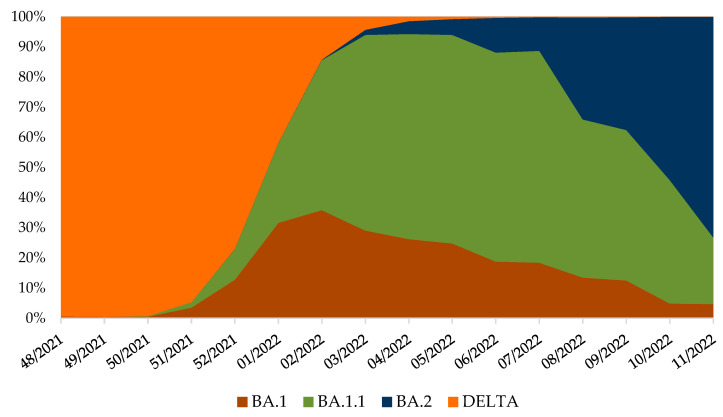
Variant frequencies in Croatia, from week 48/2021 until 11/2022, normalised to 100% [21,22,23,24]. SARS-CoV-2 positive samples were collected through routine genomic monitoring of SARS-CoV-2 in collaboration with ECDC [28] and uploaded to the GISAID database [21,22,23,24].

**Figure 3:**
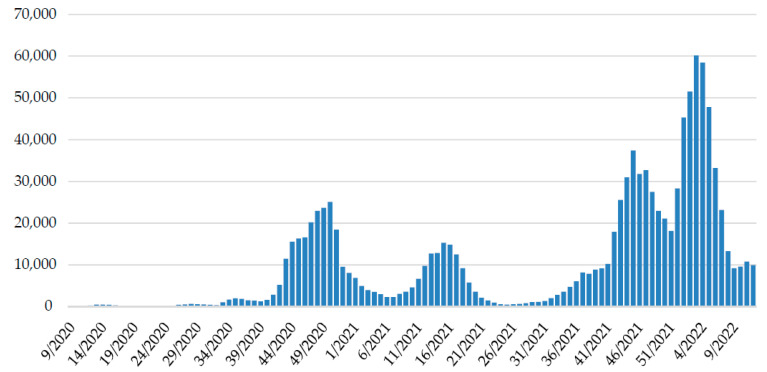
The weekly trend of SARS-CoV-2 positive cases in Croatia since the beginning of the epidemic. Data on positive patients are collected daily from all testing facilities through the central platform for COVID-19 testing registration [29] by the Croatian Institute of Public Health and reported on a website [30].

Continued genomic surveillance of SARS-CoV-2 in Croatia revealed the rapid introduction of the Omicron variant and depletion of the Delta variant (Figure 2), which resulted in the fifth pandemic wave (Figure 3).

## 3. Discussion

WGS analysis is crucial in identifying changes in viral genes that could lead to a virus profile with a significant positive impact on viral spreading or virulence [11]. Early detection of such variants is a critical step in the decision-making process of implementing measures to control viral dissemination [31].

Since February 2021, Croatia has monitored novel or emerging viral variants by WGS according to the European Centre for Disease Prevention and Control (ECDC) guidance. The ECDC suggests tracking the relative proportion of these variants throughout time and recommends the sample size required to estimate the 95% confidence interval for the proportion of a certain circulating variant when its proportion reaches 2.5% with a relative precision of 50% based on a representative selection of samples [32]. All obtained sequences are uploaded to the Global Initiative on Sharing All Influenza Data database (GISAID) [21,22,23,24].

In 2021, the introduction of different VOCs had significantly affected the number of new COVID-19 cases. Namely, the appearance of the Alpha variant (Phylogenetic Assignment of Named Global Outbreak (PANGO) lineage B.1.1.7) [18,19,20] in the population in February 2021 announced the beginning of the third epidemic wave, while the presence of the Delta variant (PANGO lineage B.1.617.2) [18,19,20] in June 2021 led to the fourth epidemic wave. Until the end of August 2021, the Delta variant with its sub-lineages [18,19,20] constituted more than 98% of all SARS-CoV-2 positive samples, while the Beta and Gamma variants were detected sporadically and connected with travellers to areas with a high prevalence and their contacts [33].

Until 30 March 2022, there have been more than 1,097,400 confirmed COVID-19 cases and 15,560 COVID-19-related deaths in Croatia [34]. At the beginning of October, the fourth pandemic wave was triggered by the dominant Delta variant, reaching a peak in week 45/2021 with a slight but steady decrease in new infections [30]. The effect of Omicron occurrence on this dynamic was noticeable soon after we detected the first Omicron positive patient, as positive cases started to rise in the last week of 2021, reaching a peak in the third week of 2022.

Although possessing a valid European Union Digital COVID certificate [35] was required to enter the venue, this outbreak was not prevented. The measures helpful in controlling the spread of the Delta variant proved to be inadequate to prevent the introduction or to control the further spread of the Omicron variant. Fortunately, most cases related to the workshop had mild flu-like symptoms after a short incubation period of 1–3 days or were fully asymptomatic.

Rapid detection of the first Omicron cases resulted from Croatia’s comprehensive and timely testing capacity and surveillance. Only ten days after the publication of the first isolated Omicron variant [3] in South Africa, we detected the first Omicron positive sample in Croatia. Under the circumstances, Croatia has implemented increasingly stringent non-pharmaceutical restrictions [36,37,38]. The combination of rapid detection with contact tracing, isolation, and quarantine measures has slowed down the outbreak’s spread in the short term. Nevertheless, the implemented stringent control measures did not interrupt the spread of the Omicron VOC in Croatia.

Genomic surveillance was crucial for the early detection of the presence and monitoring of the rapid spread of Omicron VOC. Thermo Fisher TaqPath [25] assay as a screening method for Omicron VOC was important when the Delta variant dominated, detecting potential Omicron cases in the early phase of the Omicron wave and delaying the spread of this VOC. The benefit of this screening method diminished with the introduction of the BA.2 sub-lineage in Croatia (GISAID accession ID: EPI_ISL_9148491.2) [21,22,23,24], as this sub-lineage lacks the HV69/70 deletion [39], which causes “the S gene target failure,” characteristic of the BA.1 lineage when using this assay [40]. The high transmissibility of the Omicron VOC resulted in the rapid replacement of the Delta variant by Omicron in Croatia during the first few weeks of 2022.

## 4. Materials and Methods

RNA was extracted using automated RNA extraction (GeneRotex 96, Xi’an Tianlong Science and Technology Co., Ltd., Xian, China) and qRT-PCR with Seegene Allplex™ SARS-CoV-2 Assay [41] on BIORAD CFX96 Real-Time System C1000 Thermal Cycler (Bio-Rad Laboratories, Inc., Hercules, CA, USA) was performed.

SNP assays were performed with Allplex Variant Assay I and II, Seegene [13] using a BIORAD CFX96 Real-Time System C1000 Thermal Cycler.

The reverse transcription of viral RNA was performed to obtain the viral sequence, followed by viral enrichment using the ARTIC v3 network tiled amplicon protocol [42]. The sequencing library was prepared using the Illumina DNA prep kit according to the manufacturer’s instructions. The library was sequenced using the Illumina MiniSeq sequencing system with an output of 2 × 151-bp paired-end reads. FASTQ files were uploaded on the Illumina BaseSpace hub, and FASTA files were generated using the Illumina DRAGEN COVID Lineage App version 3.5.4 with default settings. SARS-CoV-2 lineages were identified using the PANGOLIN [18,19,20,27] and GISAID [21,22,23,24] databases.

We conducted a phylogenetic analysis of sequences of interest by aligning them with the reference sequence (GISAID accession ID: EPI_ISL_402124) [21,22,23,24] using MAFFT “Multiple alignment program for amino acid or nucleotide sequences” version 7 [43]. We visualised the cladogram with the Interactive Tree Of Life (iTOL): “an online tool for phylogenetic tree display and annotation” [44]. To create an alignment, we selected and downloaded from GISAID [21,22,23,24] Croatian sequences from the Alpha and Delta lineages and worldwide sequences from Omicron lineages for the corresponding timeframe (December 2021).

Croatia performs routine genomic monitoring of SARS-CoV-2 in collaboration with ECDC. Representative and targeted positive samples have been sequenced weekly [32] since 9 February 2021 in a commercial laboratory (Eurofins Genomics Europe Shared Services GmbH).

## 5. Conclusions

The presented results highlight the importance of genomic monitoring and early detection of a novel SARS-CoV-2 variant for the timely implementation of epidemiological interventions to reduce further transmission in the population. Only ten days after the publication of the first isolated Omicron variant [3] in South Africa, using accessible knowledge of its genomic composition [4], we detected the first Omicron positive sample in Croatia and cross-border spread was almost simultaneously confirmed in Montenegro. This effective epidemiologic and genomic surveillance provided our decision-makers with the necessary inputs to implement new, Omicron-oriented, non-pharmaceutical interventions [36,37,38].

Close collaboration between epidemiologists and microbiologists in the field and the WGS team is essential to fully utilise the effect of genomic surveillance.

## Figures and Tables

**Table 1:** Single nucleotide polymorphism screening assays Ct values by targeted mutation.

	Allplex™SARS-CoV-2 Variants I Assay					Allplex™SARS-CoV-2 Variants II Assay				
Targets	E484K	HV69/70	N501Y	RdRP ^1^	IC ^2^	K417N	K417T	L452R	W152C	IC ^2^
Ct values	ND ^3^	19.74	22.52	20.20	26.34	27.11	ND ^3^	ND ^3^	ND ^3^	25.25

^1^ RdRp = RNA-dependent RNA polymerase; ^2^ IC = internal control; ^3^ ND = not detected.

**Table 2:** Demographic characteristics of patients and assigned lineages.

Country	Accession ID	Sampling Date	Age	Gender	Lineage
Croatia	EPI_ISL_7210427	2021-12-04	59	M	BA.1.17
	EPI_ISL_7892496	2021-12-04	51	M	BA.1.17
	EPI_ISL_7635857	2021-12-06	41	M	BA.1.17
	EPI_ISL_7897799	2021-12-01	37	F	BA.1.17
Montenegro	EPI_ISL_8366166	2021-12-06	52	F	BA.1.17
	EPI_ISL_8365856	2021-12-06	54	M	BA.1.17

## Data Availability

Data supporting reported results can be found in the GISAID database under corresponding GISAID Accession Numbers.

## References

[1] Tracking SARS-CoV-2 Variants. https://www.who.int/health-topics/typhoid/tracking-SARS-CoV-2-variants.

[2] (2021). Frequently Asked Questions for the B.1.1.529 Mutated SARS-CoV-2 Lineage in South Africa. NICD.

[3] Classification of Omicron (B.1.1.529): SARS-CoV-2 Variant of Concern. https://www.who.int/news/item/26-11-2021-classification-of-omicron-(b.1.1.529)-sars-cov-2-variant-of-concern.

[4] He X., Hong W., Pan X., Lu G., Wei X. (2021). SARS-CoV-2 Omicron Variant: Characteristics and Prevention. MedComm.

[5] Assessment of the Further Emergence of the SARS-CoV-2 Omicron VOC in the Context of the Ongoing Delta VOC Transmission in the EU/EEA, 18th Update. https://www.ecdc.europa.eu/en/publications-data/covid-19-assessment-further-emergence-omicron-18th-risk-assessment.

[6] Enhancing Response to Omicron SARS-CoV-2 Variant. https://www.who.int/publications/m/item/enhancing-readiness-for-omicron-(b.1.1.529)-technical-brief-and-priority-actions-for-member-states.

[7] COVID-19 Variant Dashboard (Beta). https://gis.ecdc.europa.eu/portal/apps/opsdashboard/index.html#/25b6e879c076412aaa9ae7adb78d3241.

[8] Altarawneh H.N., Chemaitelly H., Hasan M.R., Ayoub H.H., Qassim S., AlMukdad S., Coyle P., Yassine H.M., Al-Khatib H.A., Benslimane F.M. (2022). Protection against the Omicron Variant from Previous SARS-CoV-2 Infection. N. Engl. J. Med..

[9] Servellita V., Syed A.M., Moris M.K., Brazer N., Saldhi P., Garcia-Knight M., Sreekumar B., Khalid M.M., Ciling A., Chen P.-Y. Neutralizing Immunity in Vaccine Breakthrough Infections from the SARS-CoV-2 Omicron and Delta Variants. Cell.

[10] Lyngse F.P., Kirkeby C.T., Denwood M., Christiansen L.E., Mølbak K., Møller C.H., Skov R.L., Krause T.G., Rasmussen M., Sieber R.N. (2022). Transmission of SARS-CoV-2 Omicron VOC Subvariants BA.1 and BA.2: Evidence from Danish Households. medRxiv.

[11] Genomic Sequencing of SARS-CoV-2: A Guide to Implementation for Maximum Impact on Public Health. https://www.who.int/publications-detail-redirect/9789240018440.

[12] Methods for the Detection and Characterisation of SARS-CoV-2 Variants—First Update 20 December 2021. https://www.ecdc.europa.eu/en/publications-data/methods-detection-and-characterisation-sars-cov-2-variants-first-update.

[13] Blairon L., Cupaiolo R., Piteüs S., Beukinga I., Tré-Hardy M. (2021). The Challenge of Screening SARS-CoV-2 Variants of Concern with RT-QPCR: One Variant Can Hide Another. J. Virol. Methods.

[14] Seegene Inc https://www.seegene.com/assays/allplex_sars-cov-2_variants_ii_assay.

[15] Seegene Inc https://www.seegene.com/assays/allplex_sars-cov-2_variants_i_assay.

[16] Cov-Lineages. https://cov-lineages.org/global_report_B.1.1.529.html.

[17] Cov-Lineages. https://cov-lineages.org/global_report_B.1.617.2.html.

[18] Rambaut A., Holmes E.C., O’Toole Á., Hill V., McCrone J.T., Ruis C., du Plessis L., Pybus O.G. (2020). A Dynamic Nomenclature Proposal for SARS-CoV-2 Lineages to Assist Genomic Epidemiology. Nat. Microbiol..

[19] O’Toole Á., Hill V., Pybus O.G., Watts A., Bogoch I.I., Khan K., Messina J.P., Tegally H., Lessells R.R., Giandhari J. (2021). Tracking the International Spread of SARS-CoV-2 Lineages B.1.1.7 and B.1.351/501Y-V2 with Grinch. Wellcome Open Res..

[20] O’Toole Á., Scher E., Underwood A., Jackson B., Hill V., McCrone J.T., Colquhoun R., Ruis C., Abu-Dahab K., Taylor B. (2021). Assignment of Epidemiological Lineages in an Emerging Pandemic Using the Pangolin Tool. Virus Evol..

[21] GISAID—Initiative. https://www.gisaid.org/.

[22] Khare S., Gurry C., Freitas L., Schultz M.B., Bach G., Diallo A., Akite N., Ho J., Lee R.T., Yeo W. (2021). GISAID’s Role in Pandemic Response. CCDCW.

[23] Elbe S., Buckland-Merrett G. (2017). Data, Disease and Diplomacy: GISAID’s Innovative Contribution to Global Health. Glob. Chall..

[24] Shu Y., McCauley J. (2017). GISAID: Global Initiative on Sharing All Influenza Data—From Vision to Reality. Euro Surveill..

[25] TaqPath COVID-19 Multiplex Diagnostic Solution—HR. //www.thermofisher.com/tr/en/home/clinical/clinical-genomics/pathogen-detection-solutions/covid-19-sars-cov-2/multiplex.html.

[26] Kidd M., Richter A., Best A., Cumley N., Mirza J., Percival B., Mayhew M., Megram O., Ashford F., White T. (2021). S-Variant SARS-CoV-2 Lineage B1.1.7 Is Associated With Significantly Higher Viral Load in Samples Tested by TaqPath Polymerase Chain Reaction. J. Infect. Dis..

[27] COG-UK. https://pangolin.cog-uk.io/.

[28] European Centre for Disease Prevention and Control (ECDC) Methods for the detection and identification of SARS-CoV-2 variants. https://www.ecdc.europa.eu/sites/default/files/documents/Methods-for-the-detection-and-identification-of-SARS-CoV-2-variants-WHO-ECDC.pdf.

[29] Instructions for Working with the Central Platform for Registration of COVID Testing/Croatian Institute for Health Insurance. http://hzzo.hr/novosti/hzzo/upute-za-rad-s-centralnom-platformom-za-registraciju-covid-testiranja.

[30] COVID-19—Croatian Institute of Public Health-Report. https://www.hzjz.hr/aktualnosti/covid-19-izvjesce-hzjz-a/.

[31] Sequencing of SARS-CoV-2—First Update. https://www.ecdc.europa.eu/en/publications-data/sequencing-sars-cov-2.

[32] Guidance for Representative and Targeted Genomic SARS-CoV-2 Monitoring. https://www.ecdc.europa.eu/en/publications-data/guidance-representative-and-targeted-genomic-sars-cov-2-monitoring.

[33] Vilibic-Cavlek T., Stevanovic V., Brlek-Gorski D., Ferencak I., Ferenc T., Ujevic-Bosnjak M., Tabain I., Janev-Holcer N., Perkovic I., Anticevic M. (2021). Emerging Trends in the Epidemiology of COVID-19: The Croatian ‘One Health’ Perspective. Viruses.

[34] Government Official Website for Timely and Accurate Information on Coronavirus. https://koronavirus.hr/en.

[35] EU Digital COVID Certificate. https://ec.europa.eu/info/live-work-travel-eu/coronavirus-response/safe-covid-19-vaccines-europeans/eu-digital-covid-certificate_en.

[36] Decision on Temporary Prohibition and Restriction of Crossing the GP Amendments. https://www.koronavirus.hr/uploads/Odluka_granica_10_izmjena_875d7cb76a.pdf.

[37] Guidance on Treatment of Disased Patients, Their Close Contacts and Termination of Isolation and Quarantine. https://www.koronavirus.hr/uploads/Postupanje_s_oboljelima_bliskim_kontaktima_oboljelih_i_prekid_izolacije_i_karantene_1_c614d10b86.pdf.

[38] Decision on Security Measure of Temporary Ban on Entry into the Republic of Croatia. https://www.koronavirus.hr/uploads/Odluka_o_sigurnosnoj_mjeri_privremene_zabrane_ulaska_u_RH_26_11_ff2af2e93d.pdf.

[39] CoVariants: 21L (Omicron). https://covariants.org/variants/21L.Omicron.

[40] Brown K.A., Gubbay J., Hopkins J., Patel S., Buchan S.A., Daneman N., Goneau L.W. (2021). S-Gene Target Failure as a Marker of Variant B.1.1.7 Among SARS-CoV-2 Isolates in the Greater Toronto Area, December 2020 to March 2021. JAMA.

[41] Seegene Inc. https://www.seegene.com/assays/allplex_sars_cov_2_assay.

[42] Quick J. NCoV-2019 Sequencing Protocol. https://www.protocols.io/view/ncov-2019-sequencing-protocol-bbmuik6w.

[43] Katoh K., Rozewicki J., Yamada K.D. (2019). MAFFT Online Service: Multiple Sequence Alignment, Interactive Sequence Choice and Visualization. Brief Bioinform..

[44] Letunic I., Bork P. (2021). Interactive Tree Of Life (ITOL) v5: An Online Tool for Phylogenetic Tree Display and Annotation. Nucleic Acid Res..

